# Extracellular Vesicles Favor Early Peripheral Immunosenescence Through Modulation of the Senescence‐Associated Secretory Phenotype in HIV Infection

**DOI:** 10.1111/acel.70320

**Published:** 2025-12-08

**Authors:** Ricardo Cardoso Castro, Humberto Doriguetto Gravina, Fabrícia Heloísa Cavicchioli Sugiyama, Yann Lamarre, Caroline Fontanari, Bonita Powell, Olesia Gololobova, Zhaohao Liao, Fausto Almeida, Simone Kashima, Kenneth Witwer, Fabiani Gai Frantz

**Affiliations:** ^1^ Department of Biochemistry and Immunology Ribeirão Preto Medical School, University of São Paulo Ribeirão Preto São Paulo Brazil; ^2^ Department of Clinical Analyses, Toxicology and Food Science Immunology and Epigenetics Laboratory, School of Pharmaceutical Sciences of Ribeirão Preto, University of São Paulo Ribeirão Preto São Paulo Brazil; ^3^ Center for Cell‐Based Therapy, Regional Blood Center of Ribeirão Preto, Ribeirão Preto Medical School University of São Paulo Ribeirão Preto São Paulo Brazil; ^4^ Department of Molecular and Comparative Pathobiology and Biological Chemistry Johns Hopkins University School of Medicine Baltimore Maryland USA; ^5^ Department of Molecular and Comparative Pathobiology and Neurology and the Richman Family Precision Medicine Center of Excellence in Alzheimer's Disease Johns Hopkins University School of Medicine Baltimore Maryland USA

**Keywords:** extracellular vesicles, HIV infection, IL‐6, immunosenescence, miR‐21‐5p

## Abstract

HIV infection induces chronic immune activation, predisposing people living with HIV (PLWH) to early immunosenescence. It is essential to understand the mechanisms behind the senescence‐associated secretory phenotype (SASP). This study investigates the role of extracellular vesicles (EVs) in peripheral immunosenescence in HIV infection associated with SASP modulation. Biological and in silico analyses were performed to explore the crosstalk between EVs, miR‐21‐5p, and SASP. Plasma EVs from PLWH were isolated and used to stimulate peripheral blood mononuclear cells (PBMCs) to evaluate their potential to induce SASP. Mononuclear cells were transfected in vitro to induce the production of EVs carrying specific miR‐21‐5p and assess their role in SASP induction. Inflammatory and senescence markers were analyzed using an immunoassay, flow cytometry, and RT‐qPCR. Biological verifications revealed that plasma EVs from PLWH predominantly induce SASP and drive IL‐6 production in HIV‐uninfected PBMCs. We confirmed that miR‐21‐5p expression was increased in plasma EVs from chronically infected PLWH on antiretroviral therapy (ART). Furthermore, we demonstrated that EVs overexpressing miR‐21‐5p induced SASP, specifically increasing IL‐6 levels. SASP‐associated cytokines were associated with PLWH with impaired CD4 T cell recovery and a higher prevalence of CD8^+^ and CD4^+^ CD57^+^ T cells. We also corroborate that IP‐10, IFN‐γ, and IL‐6 could be potential biomarkers for identifying PLWH at greater risk of immunosenescence. Our findings provide insights into EVs driving SASP/IL‐6 release, and this effect becomes more evident in EVs that carry miR‐21‐5p. This finding highlights a potential mechanism by which EVs and IL‐6 contribute to peripheral immunosenescence in HIV‐uninfected cells.

## Graphical abstract

The senescence‐associated secretory phenotype (SASP) is one of the main factors contributing to immunosenescence in chronic HIV infection. Extracellular vesicles (EVs) present in the plasma of chronically infected people living with HIV (PLWH) represent an important mechanism for inducing SASP, particularly IL‐6 expression in peripheral cells. This modulation of SASP via EVs may be mediated by the transport of miR‐21‐5p. SASP‐associated cytokines, such as IP‐10, IFN‐γ, and IL‐6, could serve as potential biomarkers to identify individuals with immunosenescence.

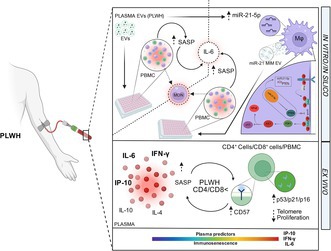

## 1 Introduction

HIV immunopathogenesis mainly affects T cells, monocytes, and macrophages, which are central components of the immune system and, therefore, suffer direct and indirect impacts from the infection (Collins et al. 2021; Tolomeo and Cascio 2024; Muñoz‐Muela et al. 2024; Woottum et al. 2024). Consequently, a significant proportion of patients are progressing to chronic inflammation. This state of inflammation is influenced by many factors, including the persistent activation of immune system cells, resulting in the secretion of cytokines, chemokines, and inflammatory mediators (Lichtfuss et al. 2011).

Many studies have shown that this persistent inflammatory profile can favor the process of cellular dysfunction and consequently lead to immunological senescence (Desai and Landay 2010; Nasi et al. 2017). The natural process of senescence is typically associated with elderly individuals; however, it has been observed prematurely in people living with HIV (PLWH), with immunological changes and age‐related illnesses, such as cardiovascular diseases and cognitive impairment (Fülöp et al. 2017; Sokoya and Steel 2017; Deeks 2011). During senescence, cells undergo a decline in proliferative capacity, attributed mainly to telomere shortening and the initiation of senescence pathways mediated by p21, p16, and p53 (Wiley and Campisi 2016). Another important factor associated with the senescence profile is that, despite losing the ability to divide, cells remain metabolically active. Pro‐inflammatory cytokines, including IL‐6, TNF‐α, and IFN‐γ, favor the senescence process and are known to be part of the senescence‐associated secretory phenotype (SASP) (Mojsilović et al. 2021). It is consistently shown that PLWH tend to have elevated plasma levels of these pro‐inflammatory cytokines (Lichtfuss et al. 2011; Ellis et al. 2020; Espindola et al. 2015). However, the underlying mechanisms driving SASP modulation in peripheral cells, especially monocytes of PLWH with detectable viral load, remain undefined, posing a critical challenge to advancing the understanding of the immunosenescence process in chronic HIV infection.

Besides the well‐documented direct effects on cell cycle disruption and inflammation disturbance of senescent cells, extracellular vesicles (EVs) generated and released by cells undergoing senescence may provoke “bystander” senescence in adjacent cells by carrying bioactive molecules such as microRNAs (miRNAs) (Takasugi 2018). EV miRNAs have been proposed as markers and drivers of disease progression, immune activation, inflammation, and oxidative stress in HIV pathogenesis (Bazié et al. 2021; Balducci et al. 2019; Hubert et al. 2015; Chettimada et al. 2020). Previous studies have shown that miR‐21‐5p is upregulated in cells, plasma, and EVs, contributing to the dysregulation of immune responses and the progression of HIV‐associated pathologies (Chettimada et al. 2020; He et al. 2021; Yelamanchili et al. 2010; Sánchez‐Del Cojo et al. 2017; Meseguer‐Donlo et al. 2023; Wu et al. 2017). Although miR‐21‐5p has been identified as abundant in EVs derived from plasma samples of PLWH undergoing antiretroviral therapy (ART), to our knowledge, there are no studies demonstrating that the transport of this miRNA can induce SASP in peripheral cells not infected by HIV.

Here, we demonstrated that EVs isolated from PLWH plasma induced the SASP profile, significantly increased IL‐6 levels in peripheral blood mononuclear cells (PBMCs) from HIV‐uninfected individuals. We confirmed that miR‐21‐5p expression was increased in EVs from the plasma of chronically infected PLWH on ART. After that, we demonstrated that miR‐21‐5p overexpressed in EVs can substantially elevate IL‐6 levels after uptake in HIV‐uninfected PBMCs. The biological effects were assessed by measuring the levels of this cytokine and various inflammatory mediators connected to the SASP, such as IFN‐γ, IP‐10, TNF‐α, IL‐10, and sCD163. These mediators were associated with impaired recovery of CD4 T cells in PLWH and an increased presence of CD8^+^ and CD4^+^ CD57^+^ T cells. We suggested that IP‐10, IFN‐γ, and IL‐6 could be used as predictors for diagnosing PLWH, who are more likely to develop early immunosenescence. Our results emphasize that immunosenescence in PLWH can be actively shaped by regulatory components transported by EVs, such as miR‐21‐5p, offering an understanding of the dynamic nature of immune aging in HIV non‐infected cells and the importance of monitoring SASP‐associated changes and phenotypes in peripheral cells to assess the prognosis of PLWH.

## 2 Methods

### 2.1 Ethics and Blood Samples Collection

The study was approved by the ethics committees of the following institutions: School of Pharmaceutical Sciences of Ribeirão Preto (CAAE: 06825018.2.0000.5403), Clinical Hospital of Ribeirão Preto—USP (CAAE: 06825018.2.3001.5440), and Regional Blood Center of Ribeirão Preto (CAAE: 06825018.2.3002.5440). The study included PLWH treated at the Special Unit for Therapy of Infectious Diseases in Brazil. The uninfected control subjects (CTRL) and leukoreduction chambers for platelet donors occurred at the Regional Blood Center of Ribeirão Preto.

### 2.2 Study Groups

We included CTRL and PLWH of both sexes and between 18 and 50 years old. We verified the clinical data of the patients shown in Table 2 in the clinical record, and we matched them by age, sex, viral load, number of CD4^+^ T cells, nadir (lowest CD4^+^ T cell count), time of infection, and treatment (Table 2). All patients were chronically infected with HIV, but none of the patients had immunodeficiency syndrome (AIDS), and the absence of clinical manifestations of the co‐infections caused by other viruses, fungi, or bacteria. Individuals in the PLWH group were patients on antiretroviral therapy (ART) and without detectable viral load. Individuals with compromised CD4 T cell recovery, known as immunological non‐responders (INR), were analyzed among PLWH. The PLWH samples were categorized into two groups based on their CD4/CD8 T cell ratio: the first group (1st) included those with a CD4/CD8 ratio lower than 0.9 (PLWH CD4/CD8 <), and the second group (2nd) included those with a ratio greater than 0.9 (PLWH CD4/CD8 >). The reference value considered normal for this ratio is between 0.9 and 2.6 (Comans‐Bitter et al. 1997). PLWH with detectable viral load (VL) were designated as the PLWH VL group.

### 2.3 Peripheral Blood Mononuclear Cell (PBMC) Isolation

The tubes of collected blood were centrifuged for 10 min at 400× *g* at room temperature to separate the plasma. The cellular portion was then diluted in PBS. PBMC isolation was performed using a Falcon tube with Ficoll‐Paque PLUS (density 1.078 g/mL). After isolation, the cell precipitate was resuspended in 1 mL of RPMI 1640 culture medium, supplemented with 10% fetal bovine serum (FBS), to adjust the number of cells for subsequent procedures.

### 2.4 Monocyte‐Derived Macrophages (MDMs)

PBMCs were centrifuged at 400× *g* for 8 min at room temperature and resuspended in RPMI 1640 culture medium supplemented with 10% heat‐inactivated human AB serum, 100 U/mL penicillin–streptomycin, 2 mM L‐glutamine, 2 mM sodium pyruvate, 10 mM HEPES buffer, and 50 ng/mL recombinant human macrophage colony‐stimulating factor (M‐CSF). The cells were seeded into 6‐well plates (2 × 10^6^ cells per well) and cultured for differentiation for 4 days. Half of the medium was removed, and an equal volume of fresh medium was added. The cells were maintained for differentiation for an additional 3 days.

### 2.5 Cell Lines

THP‐1 cells were centrifuged at 400× *g* for 8 min at room temperature and resuspended in RPMI 1640 culture medium supplemented with 10% heat‐inactivated fetal bovine serum, 100 U/mL penicillin–streptomycin, 2 mM L‐glutamine, 2 mM sodium pyruvate, and 10 mM HEPES buffer. 1 × 10^6^ cells were seeded per well into 6‐well plates and treated with 100 nM phorbol 12‐myristate 13‐acetate (PMA) and cultured for differentiation for 3 days.

### 2.6 Overexpression of miRNA Mimic in THP‐1 ML and MDM Cells

After differentiation, THP‐1 and MDM cells were transfected with miRNA mimic or negative control miRNA using Lipofectamine RNAiMAX Reagent (Invitrogen/Thermo Fisher) diluted in Opti‐MEM (Gibco). Specifically, 25 or 50 nM of mirVana miRNA mimic for hsa‐miR‐21‐5p (miR‐21 MIM) (ID: MC10206; Thermo Fisher), mirVana miRNA mimic negative control #1 (miR MIM NC) (Cat #: 4464058; Invitrogen), and along with Cy3‐labeled Anti‐miR Negative Control (Cat#: AM17011; Invitrogen). Plates were then incubated at 37°C for 24 h, after which the transfection medium was removed. The plates were washed with PBS and replenished with 3 mL of RPMI 1640 medium, FBS‐free. Through fluorescence microscopy (ZOE Fluorescent Cell Imager; Bio‐Rad), the internalization of Cy3 Dye‐Labeled Anti‐miR Negative Control in transfected cells was visualized. After 24 h, the supernatant was collected and stored at −80°C for subsequent isolation of EVs. The cell pellet was added to TRIzol reagent for further molecular analysis.

### 2.7 Flow Cytometry Analysis

Antibodies were purchased from BD Biosciences, and 1 × 10^6^ fresh PBMCs (unfrozen samples) were stained with anti‐CD3, anti‐CD4, anti‐CD8, anti‐CD57, anti‐CD28, anti‐CD45RA, anti‐CCR7, anti‐CD14, anti‐CD16, anti‐HLA‐DR, anti‐CD86, anti‐Ki67, and Fixable Viability Stain (FVS) 780 (Table 1). Cells were resuspended in FVS 780 diluted in PBS and then in PBS with 2% FBS for antibody staining. After incubation, cells were centrifuged, the supernatant was discarded, and cells were fixed in formaldehyde and stored. For proliferation assays, PBMC cultures were stimulated with anti‐CD3 (BD Biosciences; Cat# 555329; RRID: AB_395736; Clone HIT3a)/CD28 (BD Biosciences; Cat# 555725; RRID: AB_396068; Clone CD28.2) and stained extracellularly (CD3, CD4, and CD8) and intracellularly (Ki67). Intracellular staining was performed using the Transcription Factor Buffer Set (BD Biosciences) following the manufacturer's instructions. Antibodies were titrated, and to optimize fluorescence compensation, polystyrene microparticles were used BD Biosciences; CompBeads Anti‐Mouse Ig, κ/Negative Control Compensation Particles Set (Cat# 552843, RRID: AB_10051478); and CompBeads Anti‐Rat and Anti‐Hamster Ig κ/Negative Control Compensation Particles Set (Cat# 552845, RRID: AB_10058522). Data were acquired with BD FACSDiva (100,000 or 200,000 events per sample), and analysis was performed using FlowJo 10.10 software.

**TABLE 1:** Anti‐human antibodies for flow cytometry.

Epitope	Fluorophore	Clone	Company	Catalog number	RRID
CD3	BB700	SK7	BD Biosciences	566575	RRID:AB_2860004
CD4	PE‐Cy7	SK3	BD Biosciences	557852	RRID:AB_396897
CD8	BV510	SK1	BD Biosciences	563919	RRID:AB_2722546
CD57	BV421	NK‐1	BD Biosciences	563896	RRID:AB_2632391
CD28	PE	CD28.2	BD Biosciences	555725	RRID:AB_396068
CD45RA	APC	HI100	BD Biosciences	550855	RRID:AB_398468
CD197	BB515	3D12	BD Biosciences	565869	RRID:AB_2744305
Ki67	AF647	B56	BD Biosciences	558615	RRID:AB_647130
CD14	FITC	M5E2	BD Biosciences	555397	RRID:AB_395798
CD16	PerCP‐Cy5.5	3G8	BD Biosciences	560717	RRID:AB_1727434
CD86	PE	IT2.2	BD Biosciences	555665	RRID:AB_396019

**TABLE 2:** Characteristics of the study population.

	CTRL	PLWH	PLWH VL
*n*	46	30	8
Age	35	34	35
	(±8.31)	(±9)	(±6)
Sex assigned at birth	M = 33	M = 21	M = 3
	F = 13	F = 9	F = 5
Viral load (copies/mL)	—	< 40 (> 70%)	> 40 (±42679)
N° of CD4^+^ T cells (cells/mm^3^)	—	695.6 (±340)	580 (±435)
Nadir	—	359 (±280)	270 (±300)
Infection time (months)	—	91.8 (±70)	110 (±90)
Treatment time (months)	—	80 (±70)	80 (±90)

*Note:* Data presented as mean ± SD.

Abbreviations: CTRL, uninfected control; F, female; M, male; *n*, number of participants; *N* ^o^, number; PLWH, people living with HIV; PLWH VL, PLWH with viral load.

### 2.8 Cytokine Detection Assay

Cytokines, chemokines, and inflammatory mediators (TNF‐α, IL‐6, IL‐8, IP‐10, IL‐10, IL‐1β, IFN‐γ, IL‐4, IL‐17A, IL‐2, GM‐CSF, IL‐12/IL‐12p70, soluble CD163, and soluble CD14) were evaluated in plasma samples obtained from CTRL, PLWH, and PLWH VL as well as in culture supernatant. Detection assays were conducted using a multiplex immunoassay platform with magnetic beads coated with labeled antibodies, employing the Human Premixed Multi‐Analytes Kit, LXSAHM‐12 (R&D Systems), analyzed on a plate reader Luminex Multiplexing Instrument (EMD Millipore, Luminex Corporation) following the manufacturer's instructions. The concentration of IL‐6 in the supernatant culture was determined using an enzyme‐linked immunosorbent assay (ELISA MAXTM Deluxe Set, BioLegend), following the manufacturer's instructions. Soluble CD163 and CD14 levels were quantified using an enzyme‐linked immunosorbent assay (Duo Set ELISA, R&D Systems), following the manufacturer's instructions.

### 2.9 Separation of Extracellular Vesicles

Plasma‐derived EVs were isolated using sequential centrifugation (SC) protocols described by Théry et al. (2006) with adaptations (Théry et al. 2006). Briefly, plasma was prepared for EV isolation involving pre‐centrifugations at 400× *g* and 1000× *g* for 10 min to remove cells and cellular debris in the pellet. Subsequently, the supernatant was collected, and SC was conducted at 21817× *g* for 20 min at 4°C, followed by discarding the supernatant and resuspending the pellet in PBS. This process was repeated three times. Finally, the supernatant was discarded, and the EVs present in the pellet were resuspended in 100 μL of PBS and stored at −80°C. To obtain EVs produced by transfected cells in vitro, presented in culture supernatant, size‐exclusion chromatography (SEC) was performed. Culture supernatants were collected 48 h post‐transfection. Cells were removed by centrifugation at 1000× *g* for 5 min at 4°C, and the supernatant was further centrifuged at 2000× *g* for 20 min at 4°C. The supernatant was concentrated using 10 kDa MWCO Amicon filters to 0.5 mL before applying it to a qEV Original 70 nm Legacy SEC column. EV‐enriched fractions (1–4) were pooled and concentrated using 10 kDa MWCO Amicon filters to 100 μL. EVs were resuspended in 100 μL of 1× PBS. EV characterization followed the guidelines of MISEV (Minimal Information for Studies of Extracellular Vesicles) from 2018 and 2023 (Théry et al. 2018; Welsh et al. 2024).

### 2.10 Quantification of EVs

The particle concentration and size of EV preparations were measured using nanoparticle tracking analysis (NTA; NanoSight, Malvern Instruments) or the Flow NanoAnalyzer (nanoFCM, NFCM) following the guidelines of MISEV from 2018 and 2023 (Théry et al. 2018; Welsh et al. 2024).

### 2.11 Western Blot Characterization of EVs

EVs were lysed in 5× RIPA buffer (Cell Signaling Technology) with a protease inhibitor cocktail for 30 min at room temperature, then heated at 95°C for 5 min with Laemmli sample buffer (Bio‐Rad). Samples were resolved using a 4%–15% Stain‐Free gel (Bio‐Rad) and transferred to a PVDF membrane (Invitrogen) using the iBlot 2 system (Invitrogen). Membranes were probed with primary antibodies in PBS, 0.05% Tween 20, and 5% Blotting‐Grade Blocker (Bio‐Rad) for 16 h at 4°C. Primary antibodies included anti‐ALIX (Abcam; ab76608; RRID: AB_2042595), anti‐HSP‐70 (Cell Signaling Technology; Cat# 4872; RRID:AB_2279841), anti‐CD63 (Abcam; Cat# ab216130; RRID:AB_3076642; BD Biosciences; Cat# 556019; RRID:AB_396297), anti‐CD9 (Abcam; Cat# ab2215; RRID:AB_302894; BioLegend; Cat# 312102; RRID:AB_31490), anti‐Calnexin (Abcam; Cat# ab22595; RRID:AB_2069006), anti‐Syntenin (Abcam; Cat# ab133267; RRID:AB_11160262), and anti‐Cytochrome C (Cell Signaling Technology; Cat# 4272; RRID:AB_2090454). After washing, membranes were incubated with secondary antibodies anti‐mouse IgGκ BP‐HRP (Abcam; Cat# ab6789; RRID: AB_955439; Santa Cruz Biotechnology; Cat# sc‐516,102; RRID: AB_2687626) or anti‐rabbit IgG‐HRP (Cell Signaling Technology; Cat# 7074; RRID: AB_2099233; Santa Cruz Biotechnology; Cat# sc‐2357; RRID: AB_628497) for 1 h. Following washes, membranes were incubated in SuperSignal West Pico PLUS chemiluminescent substrate (Thermo Scientific) and visualized with iBright 1500FL Imager (Thermo Fisher).

### 2.12 Transmission Electron Microscopy (TEM) of EVs

Aliquots of 10 μL of EVs were adsorbed onto carbon‐coated copper grids, dried, rinsed, and stained with uranyl acetate. Images were captured using a Hitachi 7600 transmission electron microscope at 80 kV with an AMT XR80 CCD camera. SS and PS were processed similarly but with an initial 5‐min flotation and visualized using a Philips CM‐120 transmission electron microscope at 80 kV.

### 2.13 Isolation of RNA and RT‐qPCR Assays: PBMC

To evaluate gene expression, messenger RNA (mRNA) was extracted using the PureLink RNA Mini kit (Ambion, Life Technologies), and cDNA was generated using the High‐Capacity cDNA Archive kit (Applied Biosystems) following the manufacturer's instructions. The following genes were probed using qPCRBIO SyGreen Mix (PCRBiosystems) for qPCR: *p21*, *p16*, *p53*, and β‐actin (Exxtend Biotecnologia; Table S1). The sequences of the primers used are listed in the table below. The PCR cycling conditions were as follows: 95°C for 10 min, followed by 40 cycles of 95°C for 15 s and 60°C for 1 min. Results were analyzed using the 2^−ΔΔCT^ method. β‐Actin (Exxtend Biotecnologia) was used as the constitutive control. The reaction was performed using the StepOnePlus system (Applied Biosystems).

### 2.14 Isolation of RNA and RT‐qPCR Assays: THP‐1 ML, MDM, and EVs

Total RNA from cells and EVs was extracted using TRIzol LS reagent (Thermo Fisher) and the RNeasy Micro kit (QIAGEN) following the manufacturer's instructions for the protocol for adherent cells. RNA concentration and purity were measured with NanoDrop (Thermo Fisher). Reverse transcription of miRNA was performed using individual TaqMan microRNA reverse transcription kits (ABI 4366597) following the manufacturer's protocol. qPCR analysis of miRNA was conducted with 2 μL (10 ng) of RNA input from cells using TaqMan microRNA reverse transcription kits and looped stem TaqMan/qPCR probe sets (ABI 4427975): cel‐miR‐39 (ID# 000200), miR‐16 (ID# 00039), miR‐21‐5p (ID#000397), and TaqMan Universal Master Mix II, no UNG (ABI 4440040). The qPCR was run on a CFX96 Real‐Time System (Bio‐Rad) in triplicate with cycling conditions of 95°C for 10 min, followed by 45 cycles of 95°C for 15 s and 60°C for 1 min. Quantitative analysis of miR‐21‐5p expression was performed by normalizing to the endogenous control miR‐16. Relative expression levels were calculated using the 2^−ΔΔCT^ method, with the lipofectamine condition serving as the reference for fold‐change determination. For mRNA analysis, a high‐capacity cDNA synthesis kit (ABI cat#4368813), TaqMan qPCR primer probe set ABI: (cat#4331182) (*p53* ID#Hs01034249_m1, *p21* ID#Hs00355782_m1, *PTEN* ID#Hs02621230_s1, ꞵ‐actin ID# Hs01060665_g1), and TaqMan Universal Master Mix II, no UNG were used following the kit protocol. qPCR was run on a CFX96 Real‐Time System (Bio‐Rad). The qPCR was run on a CFX96 Real‐Time System in triplicate with cycling conditions of 50°C for 2 min, 95°C for 10 min, followed by 40 cycles of 95°C for 15 s and 60°C for 1 min. mRNAs were normalized to β‐actin using the 2^−ΔΔCT^ method.

### 2.15 Telomere Length Analysis

Quantitative real‐time PCR was used to assess PBMC telomere length changes with the Absolute Human Telomere Length Quantification qPCR Assay Kit (ScienCell). The telomere primer set amplifies telomere length by comparing samples with a 100‐base pair telomere sequence on human chromosome 17. PBMCs were centrifuged, and DNA was extracted using the PureLink Genomic DNA Mini Kit (Thermo Fisher). Each PCR reaction contained genomic DNA, telomere primer, qPCR master mix, and water. Real‐time PCR was performed, and average telomere length was calculated following the manufacturer's instructions.

### 2.16 EV‐Stimulated Cell Cultures

PBMCs from CTRL individuals were adjusted to obtain 5 × 10^5^ cells/well in AIM‐V or RPMI culture medium FBS‐free and then seeded into a 96‐well plate. Subsequently, cells were treated with EVs (10^9^ particles/mL) isolated from plasma or culture supernatant. The final volume in each well was 200 μL. In some experiments, lipopolysaccharide (LPS) was used as a positive control. The cells were cultured for 12 or 24 h. After the incubation period, the culture supernatant was collected for cytokine measurement, while the cell pellet was used for RNA extraction, phenotypic analysis, and flow cytometry.

### 2.17 Statistical Analysis

All analyses were performed using GraphPad Prism 9 or the R Software. Statistical analysis was chosen based on the distribution of parametric or non‐parametric data, followed by the D'Agostino‐Pearson omnibus test. The analysis was performed using an unpaired two‐tailed *t*‐test or one‐way ANOVA with Tukey's multiple comparisons test. Pearson correlations were performed in GraphPad Prism 9. Differential expression characterization in flow cytometry employed X‐shift, Flow Self‐Organizing Map (FlowSOM), and cluster explorer Uniform Manifold Approximation and Projection (UMAP) in FlowJo 10.10. Heatmap, dendrogram, and scope tree graphs were generated using FlowJo 10.10 algorithms. Analyses of cytokines, chemokines, and inflammatory mediators utilized a heatmap, dendrogram, and principal component analysis (PCA) using the self‐organizing maps (SOM) integrated approach in MetaboAnalyst 6.0.

To determine the optimal cut‐off points for diagnostic discrimination, receiver operating characteristic (ROC) curve analyses were performed using R version 4.2.3. The area under the curve (AUC) and corresponding 95% confidence intervals (95% CI) were calculated, and statistical significance was assessed using the DeLong test. AUC values were classified as follows: 0.9–1.0, excellent; 0.8–0.9, good; 0.7–0.8, regular; 0.6–0.7, acceptable; and 0.5–0.6, unsatisfactory (Mandrekar 2010). The ideal cut‐off point was determined as the one that maximized the Youden index (sensitivity + specificity—1), ensuring the best balance between sensitivity and specificity (Oehr 2025). Based on this, different cut‐off points were tested for each parameter to categorize individuals into two groups: those with a high inflammatory response (greater probability of developing peripheral immunosenescence) and those with a low inflammatory response (less likelihood of developing peripheral immunosenescence).

Differential protein expression clustering and interaction network analyses were conducted using the Reactome and STRING databases. The STRING network was performed on functional and physical protein associations, applying a high‐confidence interaction score threshold (minimum required interaction score: 0.700). To enhance the reliability of the results, we restricted the sources of interaction evidence to experimental data, co‐expression, and curated databases.

For gene expression related to senescence pathways, telomere length, and cytokine levels, the global median of each target gene was calculated, classifying individuals as “low” or “high” producers. Results were represented in diagrams, and frequencies were determined for each study group. Radar charts were created in Excel. The number of individuals varied, as detailed in the figures and legends.

## 3 Results

### 3.1 Plasma EVs From PLWH Can Induce SASP

**FIGURE 1:**
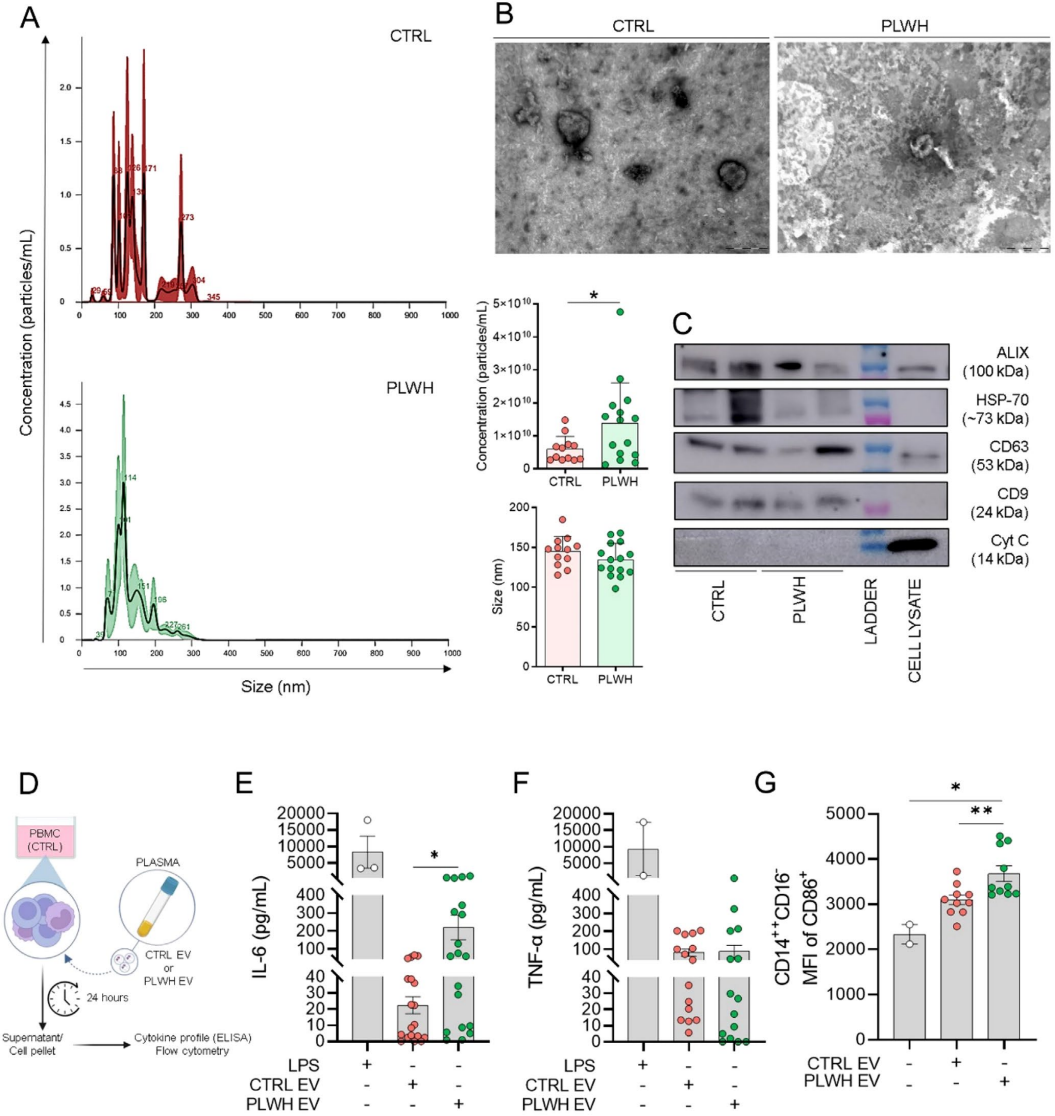
EVs isolated from PLWH plasma induce SASP in HIV‐uninfected PBMC. (A) Particle concentration and size of EVs extracted from CTRL and PLWH plasma samples. The representative graph has size/diameter (nm) on the *x*‐axis and particle concentration/mL on the *y*‐axis (left). Bar plots of average EV size (nm) and particle concentration/mL (right) in CTRL (*n* = 13) and PLWH (*n* = 15) groups. Samples were analyzed using nanoparticle tracking analysis (NTA). (B) Transmission Electron Microscopy (TEM) of EVs extracted from CTRL and PLWH plasma samples (200 nm). (C) Representative immunoblot of protein characterization of EVs extracted from CTRL and PLWH plasma. The proteins were analyzed using western blot to ALIX (100 kDa), HSP‐70 (~73 kDa), CD63 (~53 kDa), CD9 (24 kDa), and cytochrome C (Cyt C) (14 kDa). Amount of proteins per well (10 μg). (D) Experimental design of PBMCs and EVs culture. PBMCs isolated from CTRL were cultured with plasma EVs from CTRL and PLWH for 24 h. LPS was used as a positive control (10 ng/mL). Then, the supernatant was collected to evaluate the cytokine profile, and the cell pellet was used in flow cytometry to assess the expression of CD14, CD16, and CD86. Levels (E) IL‐6 and (F) TNF‐α in the supernatant of cultures treated with EVs analyzed by ELISA. (G) Mean Fluorescence Intensity (MFI) of CD86 in the classical monocytes (CD14^++^ CD16^−^). (E, *n* = 5), (F, *n* = 4), and (G, *n* = 2). The bars represent the mean ± S.E.M. of each group, and each point represents an individual. (E–F) Two data points were excluded from the LPS‐stimulated condition due to values exceeding the detection limits of the assay. Statistical analysis was performed by unpaired two‐tailed *t*‐test or one‐way ANOVA with Tukey's multiple comparisons test. ***p ≤ 0.0005, **p ≤ 0.005, *p ≤ 0.05.

To evaluate the peripheral immunomodulatory capacity of EVs from PLWH plasma, PBMCs from control individuals were cultured in vitro with EVs. We first isolated and characterized EVs, demonstrating that those derived from PLWH have an average size of 134.4 nm and a concentration of 1.38 × 10^10^ particles/mL. In contrast, EVs from the CTRL group exhibit an average size of 144.9 nm and a lower concentration of 5.99 × 10^9^ particles/mL compared to PLWH (Figure 1A). Furthermore, we conducted a characterization using TEM, demonstrating that both EV groups exhibited typical morphological characteristics (Figure 1B). Additionally, we confirmed the presence of specific surface markers for EVs, including ALIX, HSP‐70, CD63, and CD9, through Immunoblot analysis (Figure 1C).

To assess SASP induction by EVs, we treated PBMCs from CTRL for 24 h with EVs derived from either CTRL or PLWH (Figure 1D) and assessed the cytokine profile by evaluating the production in the supernatant. Surprisingly, we observed a selective increase in IL‐6 secretion in PBMC cultures exposed to EVs from PLWH, with no significant change in TNF‐α (Figure 1E,F). Furthermore, we assessed the expression of CD86, an activation receptor in monocyte subsets. EVs from PLWH lead to an increase in CD86 in the CD14^++^CD16^−^ subset (Figure 1G). These findings demonstrate that EVs from PLWH exert an inflammatory effect on monocytes, characterized by enhanced secretion of IL‐6, a cytokine associated with the SASP.

### 3.2 Transport of miR‐21‐5p via EVs Increased IL‐6 Levels In Vitro

**FIGURE 2:**
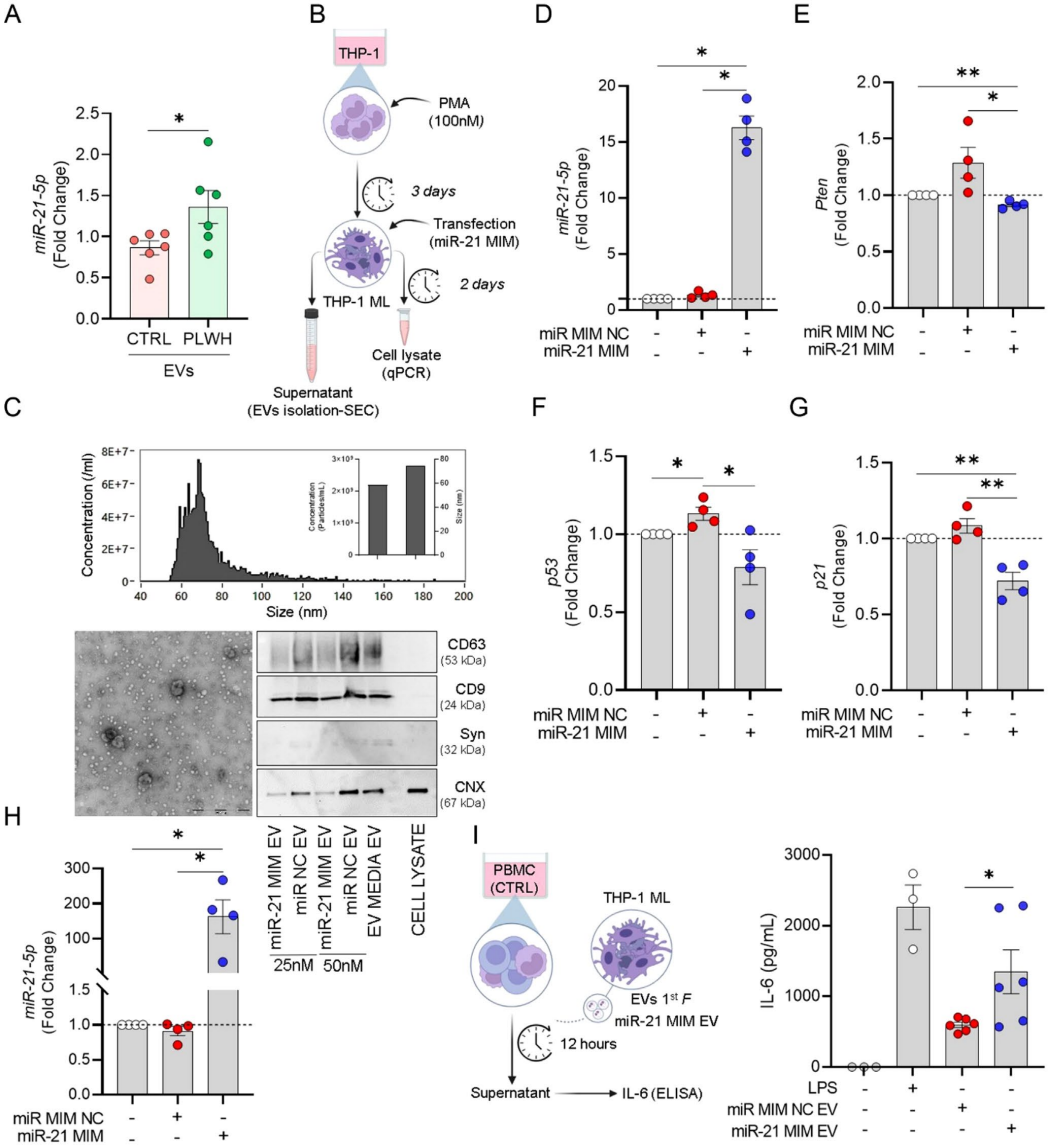
Mir‐21‐5p present in EVs is an important mechanism for inducing IL‐6 in monocytes. (A) RT‐qPCR analysis of miR‐21‐5p expression relative to EVs extracted from CTRL (*n* = 6) and PLWH (*n* = 6) plasma. (B) Experimental design of culture and transfection from macrophage‐like THP‐1 (THP‐1 ML): THP‐1 cells were seeded in 6‐well plates with RPMI complete medium supplemented with PMA (100 nM) for three days and then transfected with miR‐21‐5p mimic (miR‐21 MIM) and miRNA mimic negative control (miR MIM NC). The supernatant was collected 48 h after transfection for EV isolation by SEC, and the cell pellet was used for RNA isolation. Characterization of EVs from THP‐1 cells: (C) Particle concentration and size analysis of first (1st) fraction (top) of EVs. The representative graph has the size (nm) on the *x*‐axis and the concentration of particles/mL on the *y*. The samples were analyzed using the NanoFCM equipment. TEM of the 1st fraction of EVs. Representative immunoblot of protein characterization of EVs extracted from THP‐1 culture supernatant. CD63 (~53 kDa), CD9 (24 kDa), syntenin (Syn, 32 kDa), and calnexin (CNX, ~67 kDa) proteins from EVs were analyzed in the 1st fractions. (D) RT‐qPCR analysis of *miR‐21‐5p* expression relative to cell lysate 48 h after transfection. Gene expression of (E) *Pten*, (F) *p53*, and (G) *p21* in the cell lysate 48 h after transfection. (H) RT‐qPCR analysis of *miR‐21‐5p* expression relative to 1st EVs fraction. (D–H, *n* = 4). (I) Graphic representations of the experimental design: PBMCs were cultured individually with EV miR‐21 MIM isolated from the supernatant of the THP‐1 cells for 12 h. Quantification of IL‐6 levels. The LPS was used with a positive control (10 ng/mL). Supernatants were collected, and IL‐6 levels were assessed using ELISA (I, *n* = 3). Quantitative analysis of miR‐21‐5p expression was performed by normalizing to the endogenous control miR‐16. Relative expression levels were calculated using the 2^−ΔΔCT^ method, with the lipofectamine condition serving as the reference for fold‐change determination. β‐Actin served as the reference gene for normalizing *Pten*, *p53*, and *p21* expression. The bars represent the mean ± S.E.M. of each group. Statistical analysis was performed by unpaired two‐tailed *t*‐test or one‐way ANOVA with Tukey's multiple comparisons test. **p ≤ 0.005, *p ≤ 0.05.

It has been known that miRNA‐21‐5p plays a role in modulating the immune response in HIV infection (Chettimada et al. 2020; He et al. 2021; Yelamanchili et al. 2010; Sánchez‐Del Cojo et al. 2017; Meseguer‐Donlo et al. 2023; Wu et al. 2017). Therefore, we investigated whether the modulation of SASP, especially IL‐6, via EVs could be mediated by miRNA action. We confirmed that miR‐21‐5p is upregulated in EVs from the plasma of PLWH (Figure 2A). Then, to evaluate whether EVs carrying miR‐21‐5p can modulate SASP in recipient cells, we transfected THP‐1 cells with a miR‐21‐5p mimic (miR‐21 MIM) to induce overexpression (Figure 2B). We isolated EVs from the supernatants using a SEC protocol (Figure S1A). Analysis of particle counts and size showed an average concentration of 2.23 × 10^9^ particles/mL and an average diameter of 75 nm. TEM confirmed that the isolated EVs exhibited typical morphological characteristics (Figure 2C). We performed immunoblotting to detect specific surface markers for EVs, including CD63, CD9, and syntenin (Syn) (Figure 2C). Notably, these proteins showed enrichment in the first fraction compared to the second fraction (Figures 2C and S1B). Gene expression analysis revealed the presence of *miR‐21‐5p* in both the cell lysate and the first fraction of EVs, confirming the efficiency of transfection (Figure 2D,H). Interestingly, when we analyzed the predicted target genes of miR‐21‐5p, we observed that transfected cells showed a significant decrease in the expression of *PTEN* and genes related to senescence, *p53*, and *p21* (Figure 2E–G). Subsequently, we investigated the ability of EV miR‐21 MIM derived from THP‐1 cells to modulate the SASP. To assess this, we analyzed the levels of IL‐6. Notably, cultures treated with EV miR‐21 MIM exhibited an increase in IL‐6 levels (Figure 2I), suggesting an important potential role of EVs as transporters of miR‐21‐5p in regulating the inflammatory response associated with SASP.

We obtained the same response profile identified in PBMC cultures treated with EV miR‐21 MIM (Figure S2) by transfecting MDM cells, corroborating our hypothesis. The average concentration and diameter were 7 × 10^9^ particles/mL and 68 nm, respectively. TEM confirmed the isolated particles had typical EV morphology, and EV characterization showed enrichment of specific markers, such as CD9 and CNX, in the first fraction compared to the second fraction (Figure S2B,C). Confirming the success of the transfection, there was a notable increase in *miR‐21‐5p* expression 48 h post‐transfection in both the cell lysate and the initial fraction of EVs (Figure S2D,E). When PBMC cultures were treated with EV miR‐21 MIM derived from MDM cells, we also observed a significant increase in IL‐6 (Figure S2F).

**FIGURE 3:**
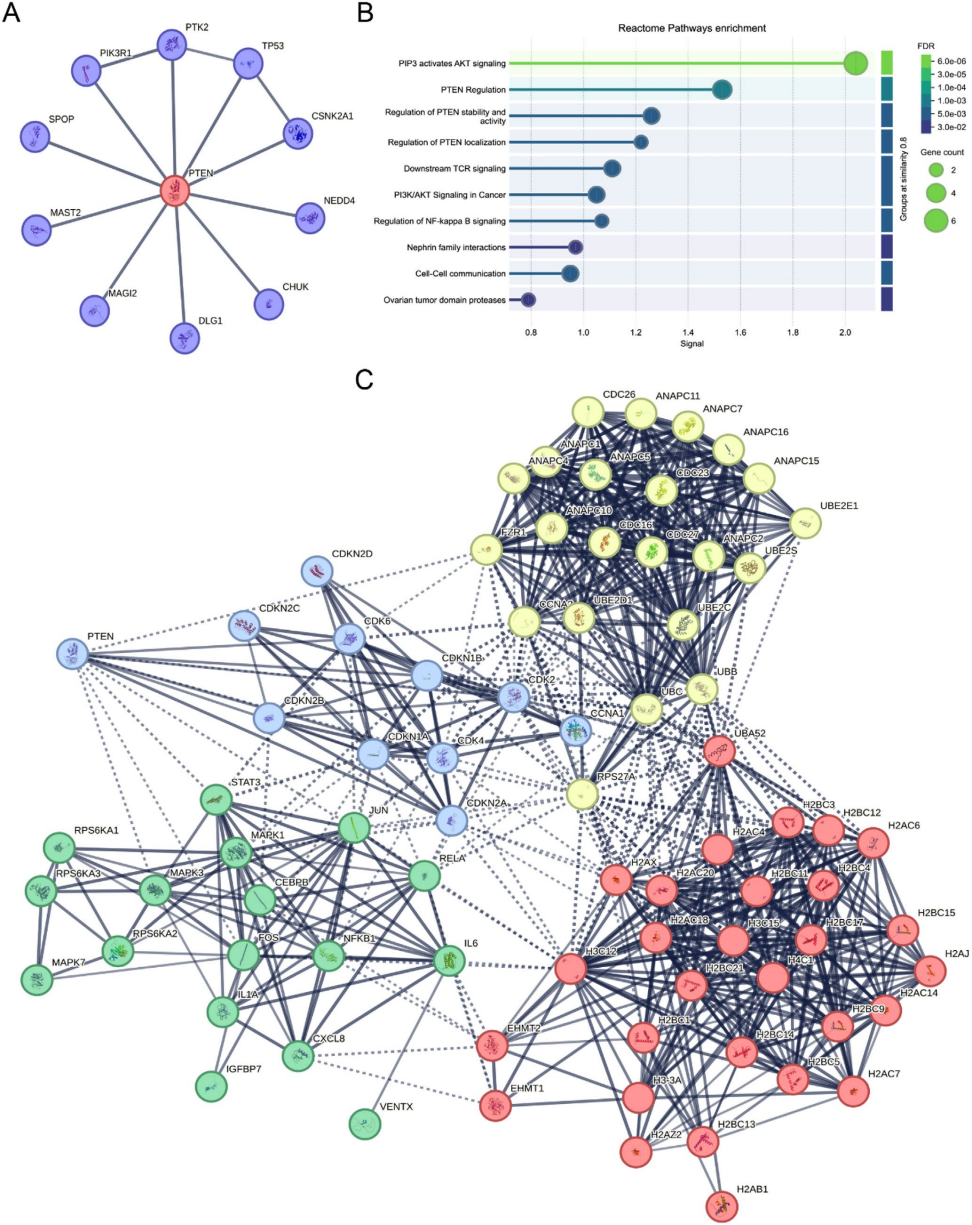
Protein–protein interaction network of PTEN and SASP‐associated factors. The network was constructed using the STRING database, highlighting PTEN as a central node interacting with key regulatory proteins. (A) The analysis includes the top 10 PTEN interactors (STRING database). (B) Functional enrichment visualization based on Reactome pathway analysis reveals the significant involvement of PTEN and its top 10 interactors in key biological processes. (C) The analysis includes PTEN interactors along with 79 proteins associated with Reactome SASP pathways. Nodes represent individual proteins, while edges indicate active interactions. The STRING network was performed on functional and physical protein associations, applying a high‐confidence interaction score threshold (minimum required interaction score: 0.700).

To propose a possible mechanism by which miR‐21‐5p modulates IL‐6 production in cells that have taken up miR‐21‐5p‐containing EVs, we investigated the protein interaction network associated with PTEN. Using the STRING database, we selected the top 10 interactors based on active interactions. The analysis revealed a well‐connected network, with PTEN as a central node interacting with key regulatory proteins, including PIK3R1, PTK2, TP53, CSNK2A1, NEDD4, CHUK, DLG1, MAGI2, MAST2, and SPOP (Figure 3A). Functional enrichment analysis using Reactome identified several biological pathways associated with PTEN and its interactors (Figure 3B). These include PIP3‐activated AKT signaling and multiple PTEN‐specific pathways such as PTEN regulation, stability, activity, and localization, reinforcing its role as a key negative regulator of PI3K/AKT signaling. Additionally, PTEN was linked to immune‐related pathways, including downstream TCR signaling and regulation of NF‐κB signaling, suggesting its involvement in T‐cell activation and inflammatory responses (Figure 3B).

To expand our understanding of SASP interactions, we conducted an additional protein interaction network analysis using STRING, selecting 79 proteins associated with Reactome SASP pathways. We observed a highly interconnected network, with direct interactions involving FZR1, UBC, CDK2, CDK6, RPS27A, CDK4, CDKN2A, CDKN1A, CDKN1B, JUN, FOS, STAT3, and MAPK3 (Figure 3C). These findings suggest a strong regulatory interplay between PTEN and SASP‐associated factors, reinforcing the hypothesis that PTEN modulates key signaling pathways linked to inflammatory responses and cellular senescence. Taken together, our findings indicate that miR‐21‐5p carried by EVs plays a crucial role in IL‐6 immunomodulation in non‐HIV‐infected peripheral monocytes. A possible mechanism involves PTEN acting as a negative regulator of PI3K/AKT signaling, potentially leading to increased NF‐κB activation and subsequent IL‐6 upregulation.

### 3.3 IL‐6/SASP Responses Drive Immune Aging in PLWH

Considering the peripheral production of IL‐6 and SASP‐associated cytokines, we investigated alterations in systemic immune response profiles linked to the senescence phenotype, with particular focus on changes observed in CD8^+^ and CD4^+^ T cells. We analyzed cytokines, chemokines, and inflammatory biomarkers to evaluate their correlation with HIV disease progression, highlighting the clinical impact of SASP in modulating immune function in T cells. We observed an increase in various pro‐inflammatory mediators in PLWH and PLWH with viral load (VL) compared to the CTRL, particularly cytokines associated with the SASP, such as IL‐6, IFN‐γ, and IP‐10 (Figure S3A). The development of a more pronounced inflammatory profile in PLWH and PLWH VL is discernible with results presented as a heatmap and dendrogram, showing the relationship between cytokines and inflammatory mediators such as IL‐6, IFN‐γ, IP‐10, GM‐CSF, and TNF‐α (Figure S3B,C). Moreover, plasma biomarkers of inflammation, sCD14 and sCD163, were elevated in PLWH VL (Figure S3D,F).

One of the main clinical parameters used to evaluate disease progression in PLWH is the CD4/CD8 T cell ratio, and senescence is associated with a decrease in this ratio (Xu and Larbi 2017). Interestingly, individuals' INR showed an increase in sCD163, IL‐6, IFN‐γ, IP‐10, TNF‐α, and IL‐10 (Figure S3E,F). These results suggest that PLWH, even with an undetectable viral load, have an altered balance of pro‐inflammatory and anti‐inflammatory plasma mediators, which may impact the peripheral immunosenescence profile.

**FIGURE 4:**
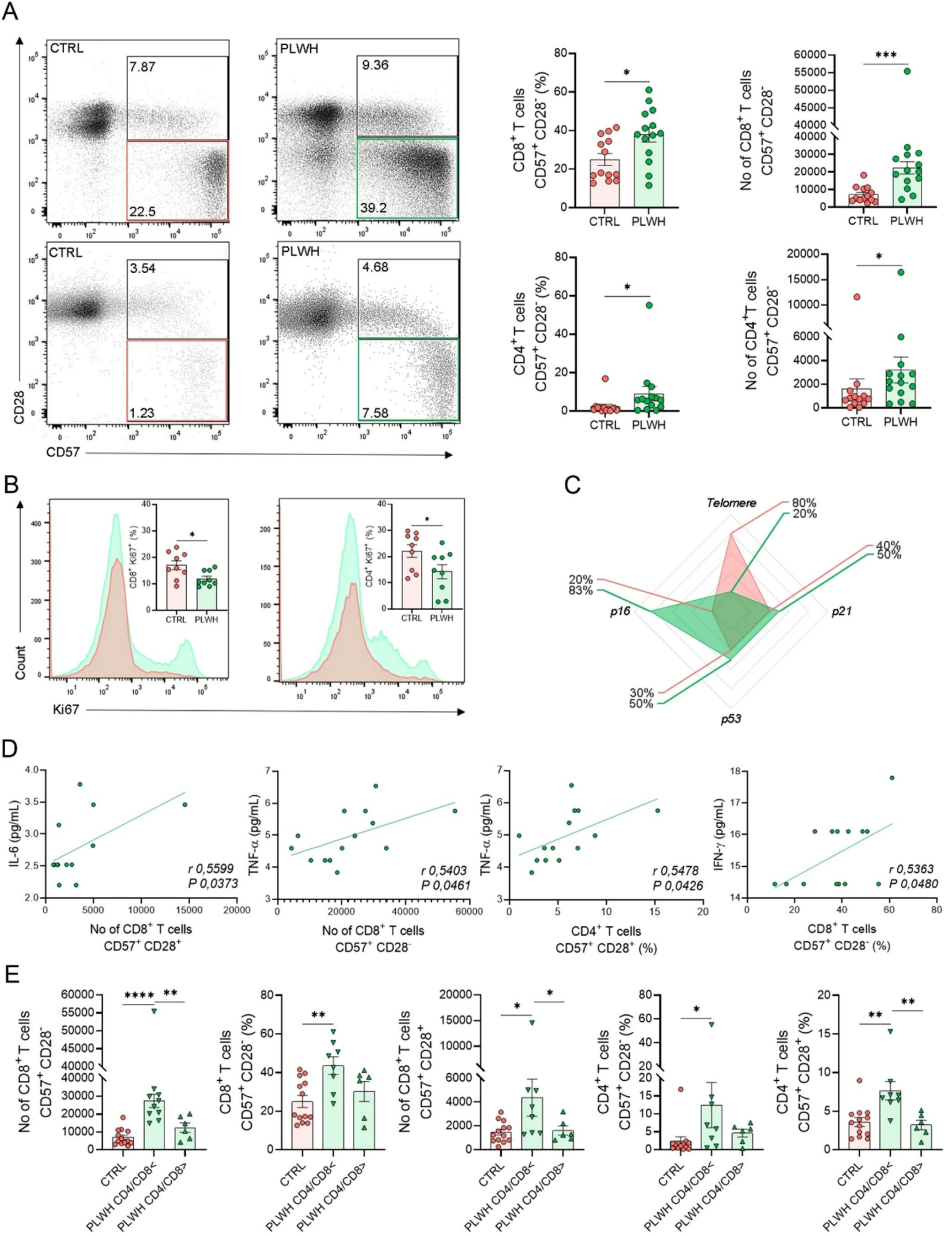
SASP is linked to altered profiles of senescent CD8 and CD4 T cells in PLWH. (A) Representative graphs of the frequency of CD8^+^ CD57^+^ CD28^+^ T cells and CD8^+^ CD57^+^ CD28^−^ T cells. Frequency and number of CD8^+^ CD57^+^ CD28^−^ T cells. Representative graphs of the frequency of CD4^+^ CD57^+^ CD28^+^ T cells and CD4^+^ CD57^+^ CD28^−^ T cells. Frequency and number of CD4^+^ CD57^+^ CD28^−^ T cells. (CTRL, *n* = 13, and PLWH, *n* = 14). (B) Frequency of CD8^+^ Ki67^+^ T cells and CD4^+^ Ki67^+^ T cells and after 5 days of stimulation with anti‐CD3/CD28. Representative histograms of one representative CTRL and PLWH (left). (C) Telomere length and *P21*, *P53*, and *P16* gene expression. Data on telomere length (CTRL, *n* = 5, and PLWH, *n* = 5) and gene expression of *P21*, *P53*, and *P16* (CTRL, *n* = 5, and PLWH, *n* = 6) were represented in the form of radar graphs, where frequencies were determined using the global median value of each target gene as a cut‐off point to determine individuals with “low” or “high” levels of a given target. (D) Positive correlation of the number of CD8^+^ CD57^+^ CD28^+^ with plasma levels of IL‐6 in PLWH. Positive correlation of the frequency of CD4^+^ CD57^+^ CD28^+^ T cells and number of CD8^+^ CD57^+^ CD28^+^ with plasma levels of TNF‐α in PLWH. Positive correlation of the frequency of CD8^+^ CD57^+^ CD28^−^ with plasma levels of IFN‐γ in PLWH. (E) Number and frequency of CD8^+^ CD57^+^ CD28^−^ T cells, number of CD8^+^ CD57^+^ CD28^+^ T cells, frequency of CD4^+^ CD57^+^ CD28^−^ T cells, and CD4^+^ CD57^+^ CD28^+^ T cells in the CTRL (*n* = 13), PLWH CD4/CD8 < (▼), (*n* = 8), and PLWH CD4/CD8 > (▲), (*n* = 6). The bars represent the mean ± S.E.M. of each group, and each point represents an individual. Statistical analysis was performed by unpaired two‐tailed *t*‐test or one‐way ANOVA with Tukey's multiple comparisons test. ****p ≤ 0.0001, ***p ≤ 0.0005, **p ≤ 0.005, *p ≤ 0.05.

Since PLWH without viremia exhibited SASP regardless of detectable viral load, we focused exclusively on the results from non‐viremic patients. Our goal is not to demonstrate the effects of active infection but to highlight the residual inflammation that persists even when the viral load is under control. This distinction is crucial for understanding the long‐term implications of HIV on immune aging and inflammation. To assess the peripheral immunosenescence profile in PBMCs from PLWH, we evaluated the expression of CD57 and CD28 on CD4^+^ and CD8^+^ T cells (Figures 4A and S4A–G). First, we observed a lower frequency and number of CD4^+^ T cells and a higher frequency and number of CD8^+^ T cells in PLWH compared to CTRL (Figure S4B,C). PLWH presented a higher frequency and number of CD4^+^ CD57^+^ CD28^−^ T cells and CD8^+^ CD57^+^ CD28^−^ T cells (Figure 4C).

To obtain a multidimensional analysis of the expression profile of these receptors in T cells, we performed high‐dimensional global mapping using flow cytometry data comprising seven parameters (CD3, CD4, CD8, CD57, CD28, CD45RA, and CCR7). For initial phenotypic characterization and identification of cell subpopulations, we employed the unsupervised clustering algorithms X‐shift and FlowSOM. UMAP projection of the data highlighted T cell populations that were preferentially enriched in PLWH compared to the CTRL group (Figure S4E). Furthermore, analysis of the median fluorescence intensity (MFI) of each marker revealed lower expression of CD4, CD28, and CCR7, and higher expression of CD8, CD57, and CD45RA in PLWH (Figure S4E). Notably, six out of the 19 clusters (clusters 9, 10, 11, 16, 18, and 19) displayed elevated CD57 expression and were more prevalent in PLWH (Figure S4F,G).

When we evaluated the proliferation of CD8 and CD4 T cells, we observed a lower proliferative capacity, characterized by the lower frequency of CD4^+^ Ki67^+^ T cells and CD8^+^ Ki67^+^ T cells in PLWH compared to CTRL (Figure 4B). Furthermore, PLWH displayed a progressive shortening of telomeres compared to the control, since upon evaluation of telomere length, only 20% of PLWH exhibited telomere lengths above the global median (high producers), while the CTRL demonstrated a frequency of 80%. Regarding the expression of genes related to senescence, 50% of PLWH were classified as high producers, with frequencies of 50% for *p53*, 50% for *p21*, and 83% for *p16* (Figure 4C).

Subsequently, we observed a correlation between SASP‐associated cytokines and CD57 expression on CD4^+^ and CD8^+^ T cells. Notably, the number of CD8^+^ CD57^+^ CD28^+^ T cells was positively correlated with IL‐6 (Figure 4D). Additionally, the frequency of CD4^+^ CD57^+^ CD28^+^ T cells showed a positive correlation with TNF‐α, while the number and frequency of CD8^+^ CD57^+^ CD28^−^ T cells were positively correlated with TNF‐α and IFN‐γ, respectively (Figure 4D). In addition, we observed that PLWH who had a ratio of CD4/CD8 T cells lower than the reference value had a higher frequency of CD4^+^ CD57^+^ CD28^+^ T cells, CD4^+^ CD57^+^ CD28^−^ T cells, a higher number of CD8^+^ CD57^+^ CD28^+^ T cells, and a higher number and frequency of CD8^+^ CD57^+^ CD28^−^ T cells (Figure 4E). Together, these data suggest that the peripheral immunosenescence profile, characterized by SASP and phenotypic and molecular alterations in CD8^+^ and CD4^+^ cells, is directly associated with the clinical prognosis of PLWH, and INR may be more susceptible to developing a response profile associated with immunosenescence.

### 3.4 Plasma Predictors to Identify PLWH Most Likely to Develop Early Immunosenescence

**FIGURE 5:**
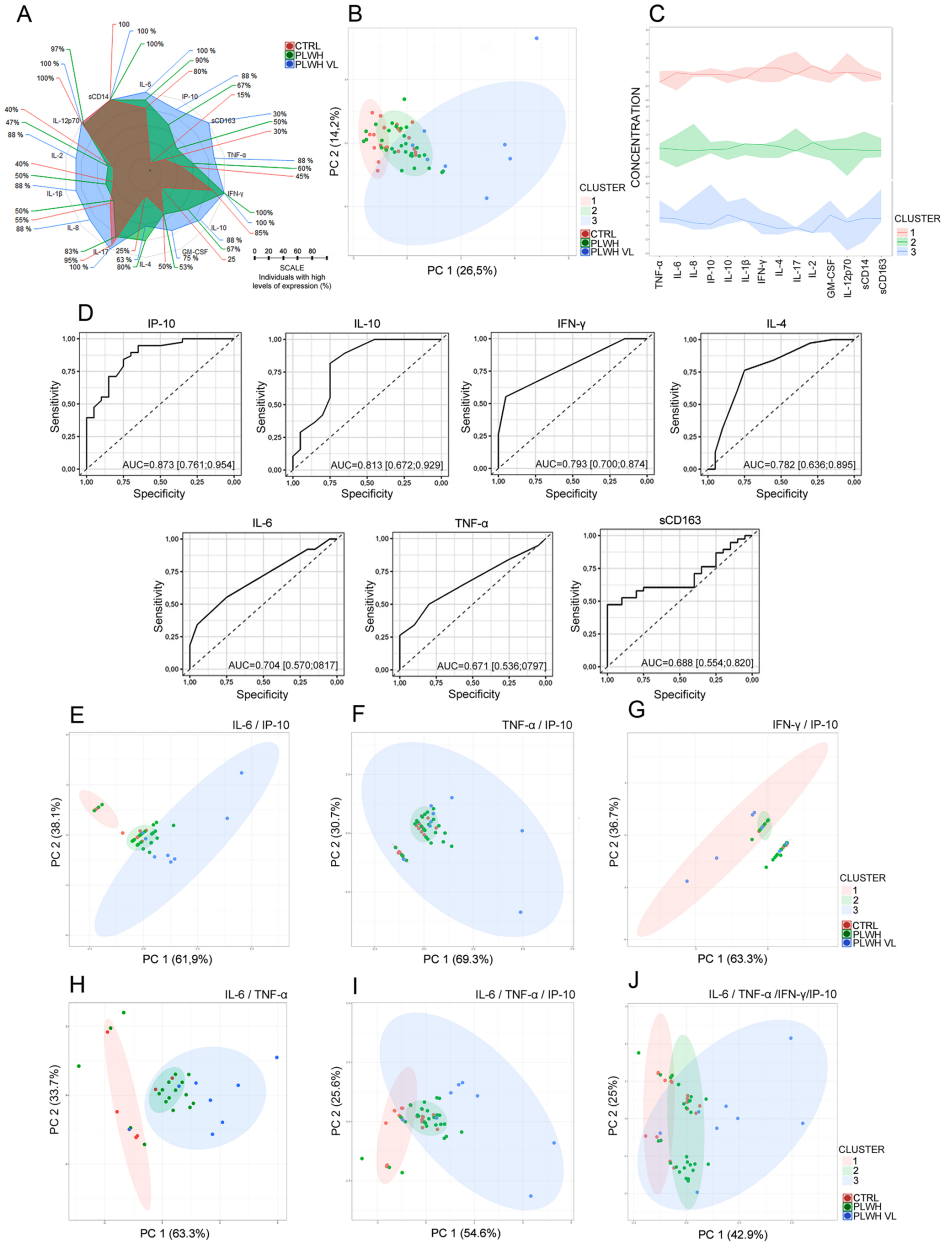
Associative and potentially predictive value of early immunosenescence. (A) Quantifying cytokine, chemokine, and inflammatory mediator levels in CTRL, PLWH, and PLWH VL plasma using the Luminex Magnetic Assay Kit or ELISA. Data were represented in radar plots, where frequencies were determined using the global median value of each target analyte as a cut‐off point to determine individuals with “low” or “high” levels of a given target. Radar charts assume that each axis shows the proportion of individuals with high target gene levels. (B) Representative plot of principal component analysis (PCA) using the integrated self‐organizing maps (SOM) approach. In this analysis, data corresponding to the levels of IL‐6, TNF‐α, IP‐10, IFN‐γ, IL‐10, GM‐CSF, IL‐4, IL‐17, IL‐8, IL‐1β, IL‐2, IL‐12p70, sCD163, and sCD14 were analyzed. The identified clusters were highlighted with different colors: Cluster 1, cluster 2, and cluster 3. (C) Representative graph of the concentration of each analyte in the different clusters. (D) ROC curve of cytokines and CRP. Univariate logistic regression analysis was performed. Performance of ROC curves of IP‐10, IL‐10, IFN‐γ, IL‐4, IL‐6, TNF‐α, and sCD163 for prediction of PLWH. CTRL (*n* = 20), PLWH (*n* = 30), and PLWH VL (*n* = 8). A representative graph of PCA analysis was used using the integrated Self‐Organizing Maps (SOM) approach. In these analyses, data corresponding to the levels of (E) IL‐6 and IP‐10, (F) TNF‐α and IP‐10, (G) IFN‐γ and IP‐10, (H) IL‐6 and TNF‐α, (I) IL‐6, TNF‐α, and IP‐10, (J) IL‐6, TNF‐α, IFN‐γ, and IP‐10 were used.

Previous results demonstrated that PLWH had marked systemic inflammation compared to CTRL individuals. Furthermore, correlation analyses showed a direct relationship between the production of specific inflammatory mediators and the prevalence of immunosenescence in PLWH. To deepen our understanding of the immunological response pattern of PLWH and identify potential marker profiles that assist in the early diagnosis of immunosenescence, we initially analyzed the frequency of individuals considered as high producers for the different analytes studied, using as a reference the global median of each mediator. Thus, in a radar graph, the state of chronic activation in PLWH is evident. The frequency of high producers was greater than 50% for all analytes evaluated in PLWH compared to the CTRL group (Figure 5A).

We applied PCA using the integrated Self‐Organizing Maps (SOM) approach, in which data corresponding to the levels of IL‐6, TNF‐α, IP‐10, IFN‐γ, IL‐10, GM‐CSF, IL‐4, IL‐17, IL‐8, IL‐1β, IL‐2, IL‐12p70, sCD163, and sCD14 were grouped (Figure 5B). Initially, the PCA generated a score plot with three clusters, considering the variation in the dataset. Even in this unsupervised model, a clear separation between the CTRL and PLWH groups is evident, with PC 1 (26.5%) and PC 2 (14.2%) (Figure 5B). The concentration graph highlights the analytes responsible for the differences between groups (Figure 5C).

Next, a ROC curve was constructed to evaluate sensitivity and specificity, as well as determine the ideal cut‐off points for each analyte, to diagnose PLWH with a predisposition to develop early immunosenescence. Using univariate logistic regression analysis, we identified IP‐10, IL‐10, IFN‐γ, IL‐4, IL‐6, TNF‐α, and sCD163 as predictive targets (Figure 5D). It is interesting to note that the ROC analysis algorithm presented significant AUC values (AUC = 0.873, 0.813, 0.793, 0.782, 0.704, 0.671, and 0.688, respectively).

Using PCA again, we evaluated combinations between specific analytes, considering the main target identified by univariate analysis, IP‐10, and its association with SASP‐related cytokines, such as IL‐6, TNF‐α, and IFN‐γ. The PCA analyses generated score plot, again identifying three clusters, considering the variation in the data set, and better segregation between the groups was observed (Figure 5E–J). The interaction between IP‐10, IL‐6, TNF‐α, and IFN‐γ mainly promoted segregation between the CTRL and PLWH groups (Figure 5E–J).

Finally, we classified individuals by applying the ideal cut‐off points established by the ROC curve algorithm for the following measurements: IP‐10 (CTRL: 35%/PLWH/PLWH VL: 95%), IL‐6 (CTRL: 25%/PLWH/PLWH VL: 55%), TNF‐α (CTRL: 15%/PLWH/PLWH VL: 50%), and IFN‐γ (CTRL: 5%/PLWH/PLWH VL: 55%). These results suggested an association between elevated cytokine levels and the development of immunosenescence in PLWH. Among these cytokines, especially IP‐10, IFN‐γ, and IL‐6 can be used as predictors for diagnosing PLWH who are more likely to develop early immunosenescence, validating that samples from the high‐inflammatory cluster also display elevated SASP‐related cytokines and monocyte activation markers, which together reinforce the biological plausibility of the senescence‐associated phenotype.

## 4 Discussion

Despite the benefits of ART in reducing circulating viral load, the chronic nature of HIV infection brings several challenges, including persistent immune activation (Kamat et al. 2012; So‐Armah et al. 2016; Lv et al. 2021; Espindola et al. 2015). Understanding the mechanisms that promote the uncontrolled production of cytokines is crucial due to their impact on HIV pathogenesis. Here, we demonstrate that plasma EVs isolated from PLWH promoted the induction of the SASP profile, leading to a significant increase in IL‐6 levels in PBMCs from uninfected individuals in a microRNA‐dependent manner. Previous data showed that plasma EVs from PLWH promoted macrophage activation, leading to increased production of inflammatory cytokines such as IL‐6, IL1‐β, and TNF‐α (Duette et al. 2018). Furthermore, we identified that the effect of these EVs specifically on circulating monocytes, key cells that are directly impacted by these EVs and play a fundamental role in maintaining systemic inflammation and SASP production via IL‐6.

It is known that EVs are essential for promoting cell–cell communication and that they can transport a variety of biological constituents, such as miRNAs (Kowal et al. 2016; Takasugi 2018). Specifically, miR‐21‐5p has been studied and identified in the inflammatory context, including in HIV infection. Although miR‐21‐5p has been previously identified in EVs from patients on ART, the test cohort and validation cohort presented mean ages of 55 and 57 years, respectively. Interestingly, we demonstrated that miR‐21‐5p is upregulated in EVs from the plasma of PLWH chronically infected, on ART, and with an average age of 34 years. This is important since we minimized the bias of natural senescence, which has already shown that miR‐21‐5p expression can increase with age (Accardi et al. 2022).

Here, we demonstrated that miR‐21‐5p overexpressed in EVs can induce a substantial elevation in IL‐6 levels after uptake in HIV‐uninfected PBMCs. These data suggest a potential pathway through which HIV‐uninfected cells may be indirectly influenced, leading to an inflammatory SASP‐related profile mediated by EVs and miRNAs in monocytes. We demonstrated that cells overexpressing miR‐21‐5p present a significant decrease in *PTEN* expression. PTEN is an important tumor suppressor gene considered a target of miR‐21‐5p, and it acts as an antagonist of the PI3K/AKT signaling pathway (Lee et al. 2018). This pathway is central to the modulation of cellular metabolism, proliferation, and longevity, in addition to activating other signaling pathways, such as the NF‐κB pathway, which is essential for the production of pro‐inflammatory cytokines, including IL‐6 (Bai et al. 2009; Liu et al. 2020). In addition, we observed that cells overexpressing miR‐21‐5p exhibit a decrease in the expression of the *p53* and *p21* proteins. PTEN plays an important role in the positive regulation of p53 activity by inhibiting the PI3K/AKT pathway, which normally promotes p53 degradation after its activation (Minami et al. 2016). Based on these findings, we can indirectly infer that overexpression of miR‐21‐5p in macrophages may reduce cellular senescence by making them metabolically more active, possibly through activation of the AKT pathway. Consequently, one potential mechanism that may explain the increase in IL‐6 in HIV‐uninfected cells that internalize EVs containing miR‐21‐5p could be associated with the PTEN/PI3K/AKT/NF‐κB axis; however, further investigation into this mechanism is necessary.

Considering the immunomodulatory effect of EVs, especially those carrying miR‐21‐5p, their biological impact becomes clearly relevant in the clinical setting of patients, since we confirmed this by observing elevated levels of various inflammatory cytokines and chemokines in the plasma of PLWH with undetectable viral load. Pro‐inflammatory cytokines such as IL‐6, TNF‐α, and IFN‐γ are described as important molecules that promote the process of senescence and are known to be part of the SASP (Mojsilović et al. 2021). In this study, we identified that IP‐10, IL‐6, and IFN‐γ could be used not only as indicators of inflammation but also as predictors of early immunosenescence in PLWH with undetectable viral load. We identified a prevalence of several of these markers in non‐responsive PLWH, such as a significant increase in plasma levels of TNF‐α, IL‐6, IP‐10, IFN‐γ, IL‐10, sCD163, and CD57 expression in CD8^+^ and CD4^+^ T cells. This suggests that such individuals may be more susceptible to the development of peripheral immunosenescence.

During senescence, cells decrease their proliferative capacity; however, they can remain metabolically active (Gey and Seeger 2013). Previously, in another cohort, it was shown that T cells in advanced stages of differentiation exhibited high expression of CD57 and CD45RA, as well as reduced expression of the co‐stimulatory molecule CD28 and the absence of the chemokine receptor CCR7 (Kared et al. 2016). Additionally, we have demonstrated that multidimensional cytometry analyses of the expression profile of these receptors showed that PLWH also had higher expression of CD45RA and lower expression of CCR7. Supporting our findings, higher expression of CD57 in CD8^+^ and CD4^+^ T cells has been associated with terminally differentiated cells and immunosenescence in PLWH (Papagno et al. 2004; Elias Junior et al. 2024; Brenchley et al. 2003; Méndez‐Lagares et al. 2013; Deeks et al. 2012). Corroborating this, PBMC from PLWH presented higher expression of *p53*, *p21*, *p16*, along with telomere shortening, closely linked to the cellular senescent profile (Kumari and Jat 2021; Deeks et al. 2012). These findings underscore the importance of initiating ART soon after HIV diagnosis and carefully monitoring the immune response during treatment. This not only aims to reduce circulating viral load but also minimizes long‐term damage to the immune system.

Our study had some limitations, including the limited number of participants. Additionally, due to sample constraints, some experiments had a reduced sample size. Regarding the EV experiments, we could not identify the vesicle origin since they were isolated from plasma, leaving the specific cell subpopulation producing the vesicles unknown. Although we did not deeply explore the mechanisms through which miR‐21‐5p increases IL‐6, our findings and in silico data suggest the involvement of the PTEN/PI3K/AKT/NF‐κB axis in this process. Since we did not evaluate other miRNAs, we cannot rule out the possibility that, in addition to miR‐21‐5p, other miRNAs are also transported by vesicles and play a role in peripheral immunomodulation. We prioritized the use of fresh blood samples, particularly for T cell phenotypic characterization, to minimize the impact of freezing on cellular senescence characterization. While other senescence markers could complement our results, we focused on CD57, the primary marker associated with T cell senescence.

We acknowledge that EV and cytokine measurements were obtained from different individuals, which limits the establishment of a definitive causal link. Nevertheless, by exposing healthy PBMCs to plasma‐derived EVs from PLWH for 24 h, we observed a selective induction of IL‐6. This finding supports a model in which EVs act as initiators of IL‐6–driven amplification of the SASP cascade, subsequently involving TNF‐α and IFN‐γ, whose plasma levels correlate with the presence of senescent CD4^+^ and CD8^+^ T‐cell phenotypes systemically. Although limited by the low yield of plasma EVs, this design precluded parallel assays but allowed us to interpret the intrinsic pro‐inflammatory activity of EVs. Altogether, our data provide biologically consistent evidence that EV‐triggered IL‐6 may represent a plausible mechanistic bridge linking EV signaling to SASP‐associated immune aging in HIV, while acknowledging the sampling limitation of our cohort and the need for further follow‐up paired studies.

In summary, we have demonstrated that the EVs have emerged as important mediators in intercellular communication, potentially facilitating the dissemination of pro‐inflammatory signals and influencing the development of SASP during HIV infection. Thus, even under ART, EVs carrying miRNAs and other immunomodulators may represent a key mechanism mediating peripheral immune modulation. Here, we suggest a potential mechanistic pathway by which HIV infection accelerates immune aging, specifically through the modulation of pro‐inflammatory and senescence‐associated factors by the action of miR‐21‐5p. Furthermore, we highlight the importance of monitoring SASP levels, such as IP‐10, IFN‐γ, and IL‐6, as well as cellular markers associated with peripheral immunosenescence. This monitoring is essential to assess clinical progression and identify PLWH with a greater predisposition to developing premature senescence and aging‐related diseases.

## Author Contributions

All authors critically reviewed and approved the submitted version of the manuscript. R.C.C. recruited the participants, provided blood samples, performed the conceptualization, conducted experiments, data curation, and formal analysis, and wrote the original, reviewed, and edited the manuscript. H.D.G. and F.H.C.S. recruited the participants, performed the data curation and formal analysis, wrote the review, and edited the manuscript. Y.L., C.F., B.P., O.G., and Z.L. performed the methodology and formal analysis. F.A., S.K., K.W., and F.G.F. provided reagents and methods. K.W. performed the data curation, supervision, and review of the manuscript. F.G.F. performed the conceptualization, funding acquisition, resources, project administration, supervision, and data curation, wrote the original, reviewed, and edited the manuscript.

## Funding

This study was funded by the Coordination for the Improvement of Higher Education Personnel (CAPES), Financial code 001, National Council for Scientific and Technological Development (CNPq), São Paulo Research Foundation (FAPESP) (Grant 2018/15066‐0, 2019/11422‐0, 2022/04593‐5 to F.G.F. and R.C.C.), and US National Institutes of Health (NIH) through AI144997 (to K.W.).

## Conflicts of Interest

The authors declare no conflicts of interest.

## Supporting information


**Appendix S1:** acel70320‐sup‐0001‐AppendixS1.docx.

## Data Availability

Data is available on request from the authors.
