# Supplementary material for: Extracellular Vesicles Favor Early Peripheral Immunosenescence Through Modulation of the Senescence‐Associated Secretory Phenotype in HIV Infection
Source: Aging Cell. 2025 Dec 8;25(1):e70320. doi: 10.1111/acel.70320 (PMC12741232; doi:10.1111/acel.70320)
Supplement: Supplementary file 1 — Appendix S1: acel70320‐sup‐0001‐AppendixS1.docx. [file ACEL-25-e70320-s001.docx]

**Supporting Information**

**Extracellular vesicles favor early peripheral immunosenescence through modulation of the senescence-associated secretory phenotype in HIV infection.**

Ricardo Castro Castro, Humberto Doriguetto Gravina, Fabrícia Heloísa Cavicchioli Sugiyama, Yann Lamarre, Caroline Fontanari, Bonita Powell, Olesia Gololobova, Zhaohao Liao, Fausto Almeida, Simone Kashima, Kenneth Witwer, Fabiani Gai Frantz.

| **Table S1. Sequences of primers** | | | |
| --- | --- | --- | --- |
| Gene | *Forward* (5’-3’) | *Reverse* (5’-3’) |  |
|  |  |  |  |
| *p16* | GAAGGTCCCTCAGACATCCC | AAACTACGAAAGCGGGGTGG |  |
| *p21* | TCTTGTACCCTTGTGCCTCG | ATCTGTCATGCTGGTCTGCC |  |
| *p53* | CCCTCCTCAGCATCTTATCC | GTACAGTCAGAGCCAACCTCAG |  |
| *ꞵ-actin* | CCAGCCTTCCTTCCTGGGCAT | AGGGCAATGATCTTGATCTTCATT |  |


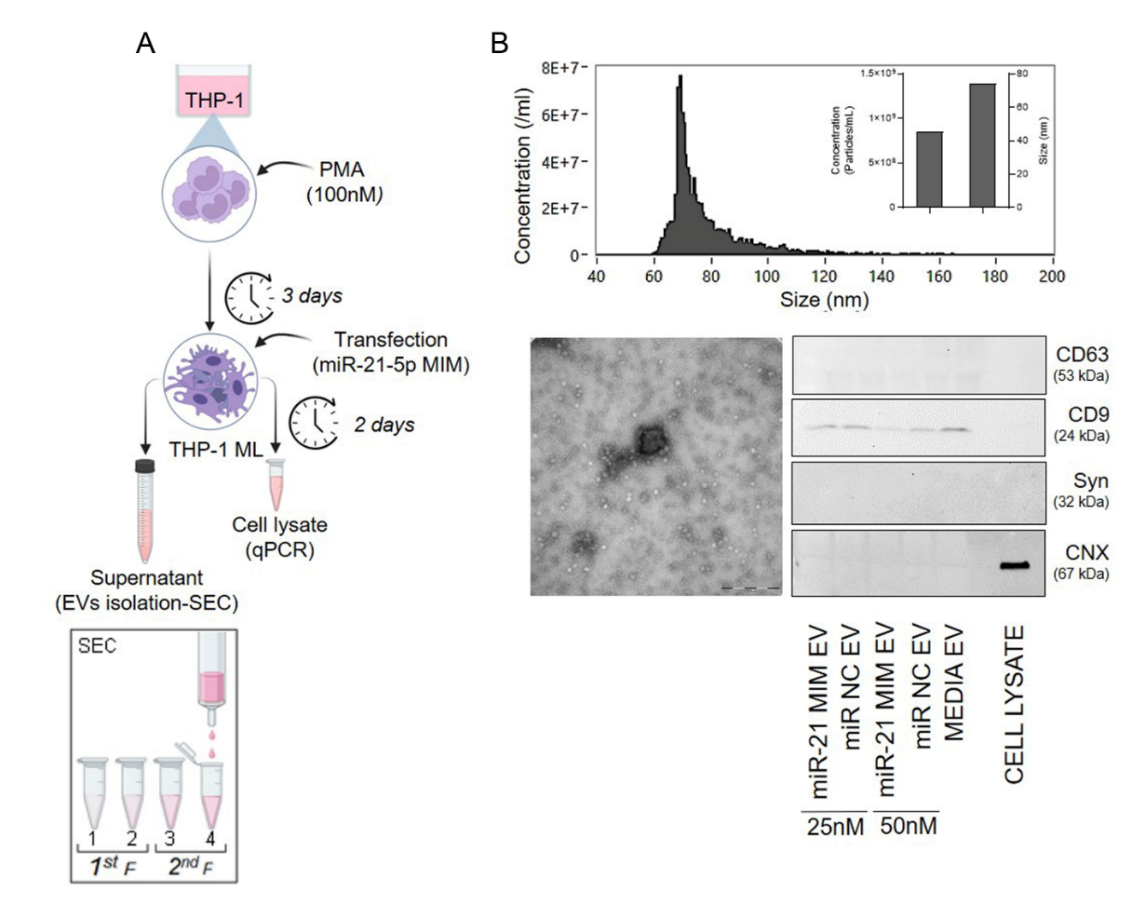


**Figure S1. Characterization of second fraction EVs Derived from THP-1 Cells. (A)** Experimental design of culture and transfection from macrophage-like THP-1 (THP-1 ML): THP-1 cells were seeded in 6-well plates with RPMI complete medium supplemented with PMA (100nM) for three days and then transfected with miR-21-5p mimic (miR-21 MIM) and miRNA mimic negative control (miR MIM NC). The supernatant was collected 48 hours after transfection for EV isolation by SEC, and the cell pellet was used for RNA isolation. **(B)** Characterization of EVs from THP-1 cells: Particle concentration and size analysis of second (2^nd^) fraction of EVs. The representative graph has the size (nm) on the x-axis and the concentration of particles/ml on the y. The samples were analyzed using the NanoFCM® equipment. TEM of the 2^nd^ fraction of EVs. Representative immunoblot of protein characterization of EVs extracted from THP-1 culture supernatant. CD63 (~53 kDa), CD9 (24 kDa), Syn (32 kDa), and CNX (~67 kDa) proteins from EVs were analyzed in the 2^nd^ fractions (right).


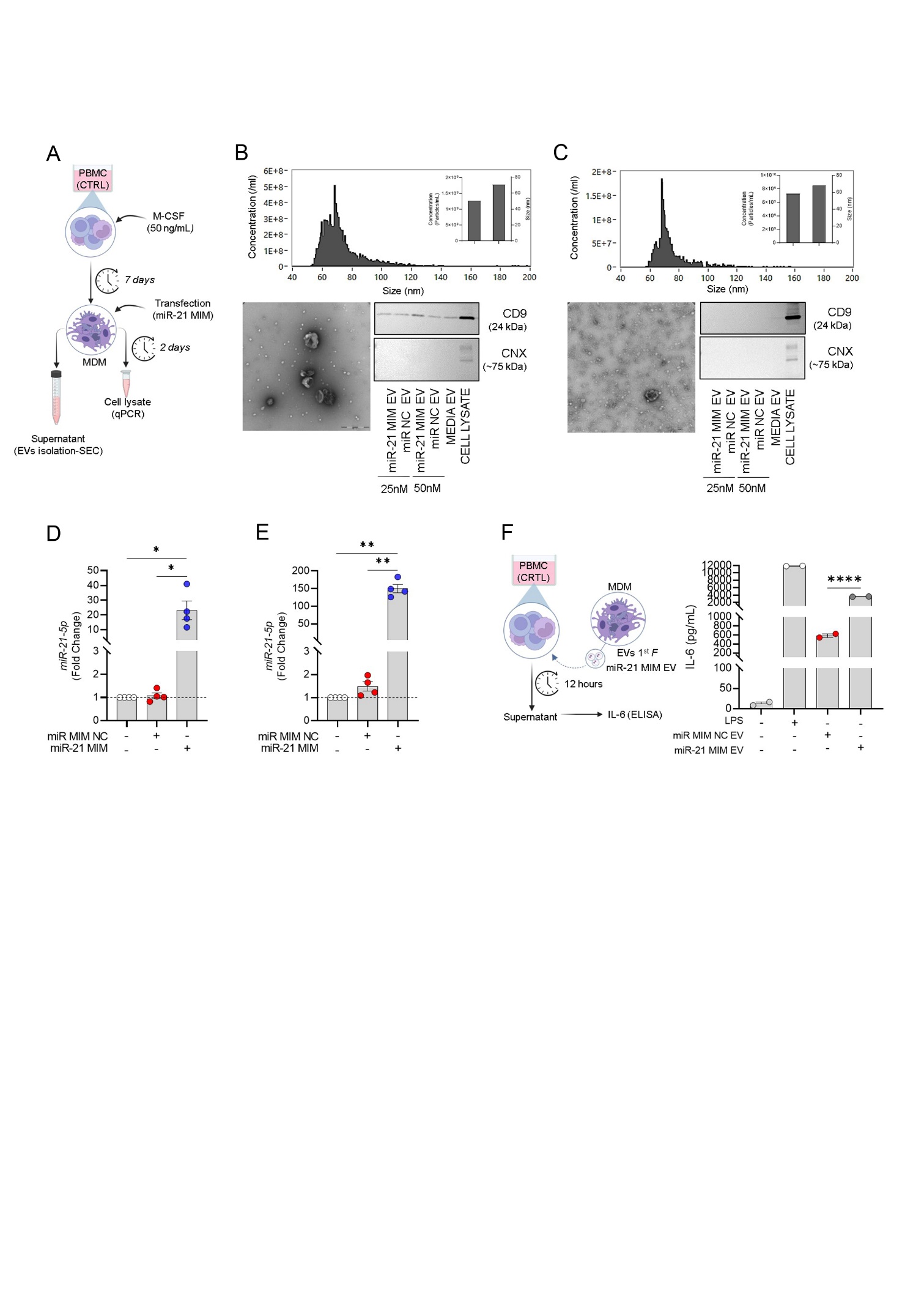


**Figure S2. miR-21-5p present in MDM EVs induced IL-6 in monocytes. (A)** Experimental design of culture and transfection from MDM. PBMCs from CTRL were seeded in 6-well plates with RPMI complete medium supplemented with M-CSF (50ng/mL) for 7 days and then transfected with miR-21-5p mimic (miR-21 MIM) and miRNA mimic negative control (miR MIM NC). The supernatant was collected 48 hours after transfection for EVs isolation by SEC, and the cell pellet was used for RNA isolation. **(B)** and **(C)** Particle concentration and size analysis of 1^st^ and 2^nd^ fractions of EVs. Particle concentration and size analysis of EVs. The representative graph has the size (nm) on the x-axis and the concentration of particles/ml on the y. The samples were analyzed using the NanoFCM® equipment. TEM of the 1^st^ fraction and 2^nd^ fraction of EVs. Representative protein gel of EVs in the 1^st^ fraction (left) and 2^nd^ fraction (right). Representative immunoblot of protein characterization of EVs extracted from MDM culture supernatant. CD9 (24 kDa) and CNX (~67 kDa) proteins from EVs. **(D)** RT-qPCR analysis of *miR-21-5p* expression relative to cell lysate 48 hours after transfection. **(E)** RT-qPCR analysis of *miR-21-5p* expression relative to 1^st^ fraction. **(F)** Graphic representations of the experimental design: PBMCs were cultured individually with EV miR-21 MIM isolated from the supernatant of the MDM cells for 12 hours. Quantification of IL-6 levels. The LPS was used with a positive control (10 ng/mL). Supernatants were collected, and IL-6 levels were assessed using ELISA. Quantitative analysis of miR-21-5p expression was performed by normalizing to the endogenous control miR-16. Relative expression levels were calculated using the 2^^−ΔΔCT^ method, with the lipofectamine condition serving as the reference for fold-change determination. The bars represent the mean ± S.E.M. of each group. Statistical analysis was performed by one-way ANOVA with Tukey’s multiple comparisons test. ****P ≤ 0.0005, **P ≤ 0.005, *P ≤ 0.05*.


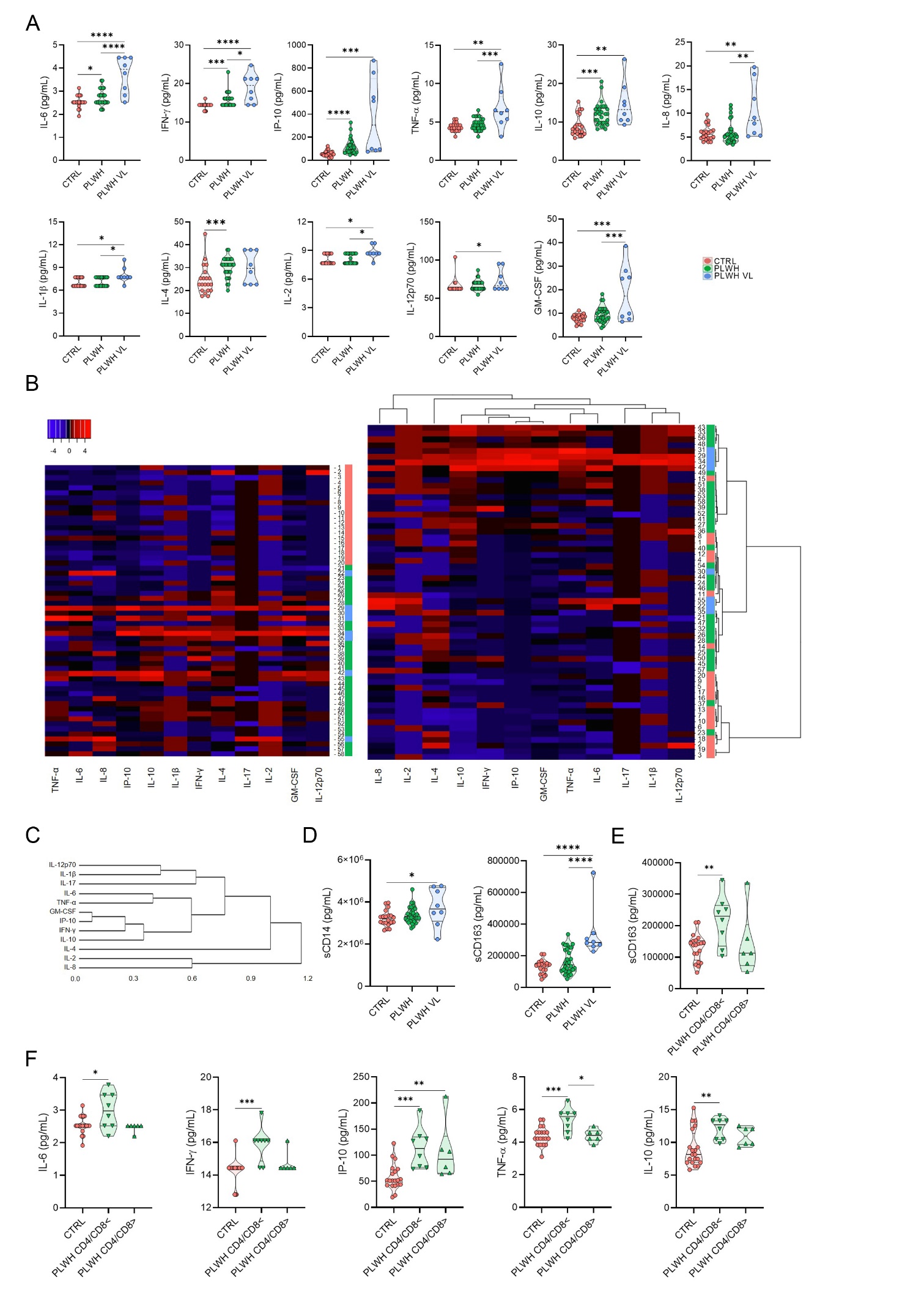


**Figure S3. Levels of Cytokines, Chemokines, and Inflammation Biomarkers in the Plasma.** **(A)** IL-6, IFN-γ, IP-10, TNF-α, IL-10, IL-8, IL-1β, IFN-γ, IL-4, IL-2, IL-12/IL-12p70, and GM-CSF levels in the plasma from CTRL, PLWH, and PLWH VL assessed using Multiplex. **(B)** In the heatmap on the left, we can see that lines 1 to 20 are composed of CTRL, and lines 21 to 58 are formed of PLWH and PLWH VL samples. In the heatmap on the right, the formation of hierarchical clusters with dendrograms was applied to rows (CTRL/PLWH/PLWH VL) and columns (cytokines/chemokines). **(C)** Graphical representation of the dendrogram of the interaction between cytokines. Furthermore, Pearson analysis was used for the distance measurement method. **(D)** The levels of sCD14 and sCD163 were evaluated in the plasma of CTRL, PLWH, and PLWH VL using ELISA. CTRL (n=20), PLWH (n=30), and PLWH VL (n=8). **(E)** sCD163, **(F)** IL-6, IFN-γ, IP-10, TNF-α, and IL-10 levels in the plasma from CTRL, PLWH CD4/CD8< (▼), and PLWH CD4/CD8> (▲). The bars represent the mean ± S.E.M. of each group, and each point represents an individual. Statistical analysis was performed by one-way ANOVA with Tukey’s multiple comparisons test. ****P ≤ 0.0001, ****P ≤ 0.0005, **P ≤ 0.005, *P ≤ 0.05*.


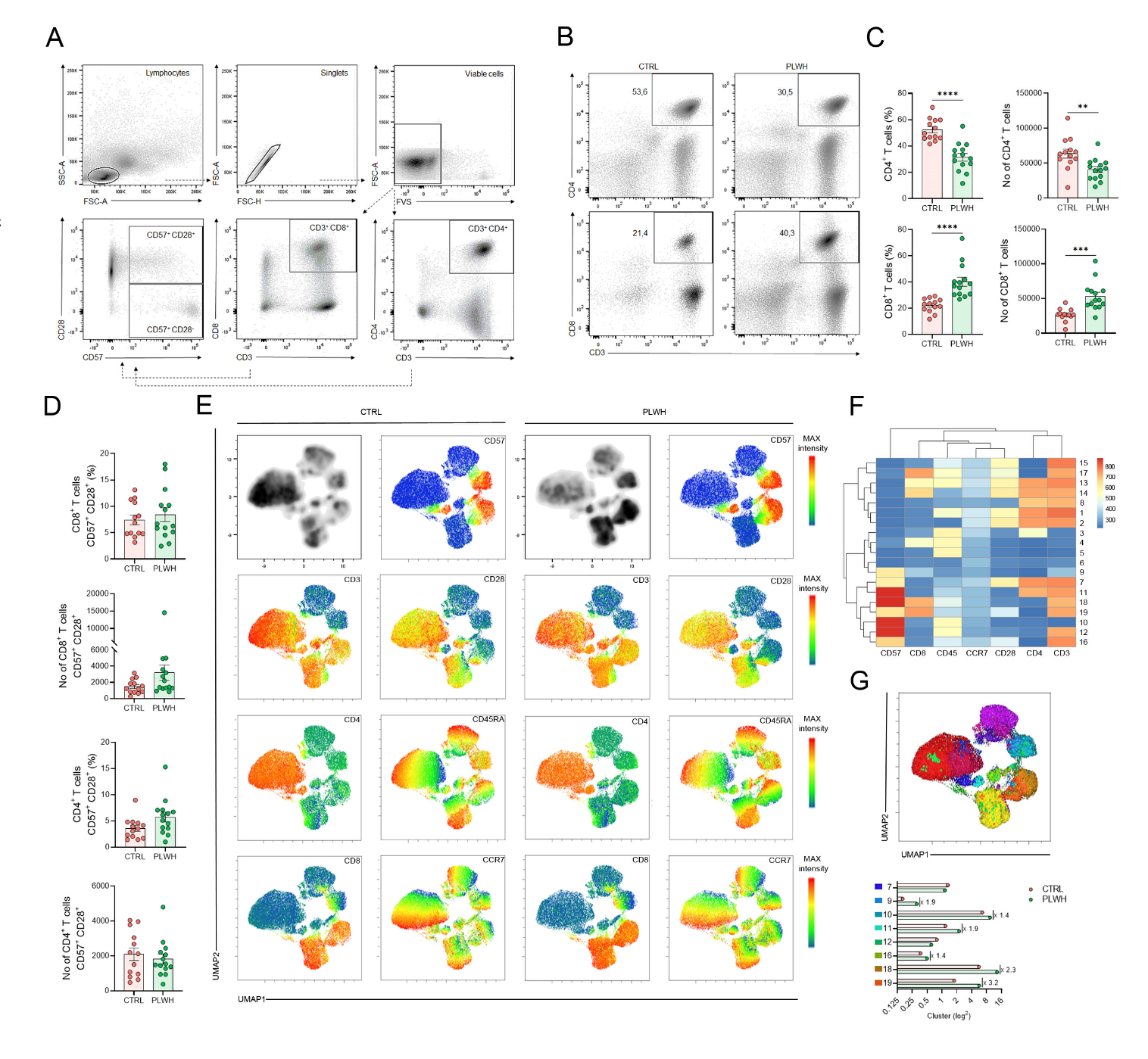


**Figure S4. CD8^+^ and CD4^+^ T cells in PLWH resemble terminally differentiated effector memory cells. (A)** Representative graphs of flow cytometry strategies used to characterize CD4^+^ T and CD8^+^ T cells expressing CD57 and CD28 in PBMCs from CTRL and PLWH. **(B)** Representative graphs of the frequency of CD4^+^ T and CD8^+^ T cells. **(C)** Frequency and number of CD4^+^ T and CD8^+^ T cells. **(D)** Frequency and number of CD4^+^ T and CD8^+^ T cells that express CD57 and CD28. **(E)** UMAP projection of T cells for all individuals grouped into the CTRL and PLWH, concatenated (10^3^ cells per sample) and overlapped (top left/density plot). Individual MFI of CD3, CD4, CD8, CD57, CD28, CD45RA, and CCR7 receptors were evaluated in UMAP projections. **(F)** MFI of CD3, CD4, CD8, CD57, and CD28 proteins as indicated in the clusters identified through FlowSOM (column-scale z-score). **(G)** UMAP projections of CD3, CD4, CD8, CD57, CD28, CD45RA and CCR7 protein expression and frequency of clusters of cells expressing higher levels of CD57 in PLWH/CTRL (Bottom). The bars represent the mean ± S.E.M. of each group, and each point represents an individual. The results were analyzed using the FlowJo analysis tool, version 10.10. CTRL (n=13) and PLWH (n=14). Statistical analysis was performed by unpaired two-tailed *t*-test. ****P ≤ 0.0001, ****P ≤ 0.0005, **P ≤ 0.005, *P ≤ 0.05*.
